# The Value of the 8th Edition of American Joint Committee on Cancer Pathological Prognostic Staging on the Selection of Postmastectomy Radiotherapy for T1–2N1 Breast Cancer

**DOI:** 10.1155/2022/7550323

**Published:** 2022-10-25

**Authors:** Jun Wang, Xiao-rong Zhong, Ting Luo, Zhong-zheng Xiang, Yuan-yuan Zeng, Tian Yang, Hong Zheng, Lei Liu

**Affiliations:** ^1^Department of Head and Neck Oncology, Department of Radiation Oncology, Cancer Center, State Key Laboratory of Biotherapy, West China Hospital, Sichuan University, Chengdu, Sichuan 610041, China; ^2^Breast Disease Center, Cancer Center, West China Hospital, Sichuan University, Chengdu 610041, China; ^3^Multi-Omics Laboratory of Breast Diseases, State Key Laboratory of Biotherapy, National Collaborative, Innovation Center for Biotherapy, West China Hospital, Sichuan University, Chengdu 610041, China

## Abstract

**Methods and Materials:**

Patients diagnosed with pT1-2N1M0 breast cancer between 2008 and 2018 in West China Hospital, Sichuan University were included. Locoregional-free survival (LRFS), distant metastasis-free survival (DMFS), disease-free survival (DFS), breast cancer-specific survival (BCSS), and overall survival (OS) were defined as endpoints. The propensity score matching (PSM), receiver operating characteristic (ROC) curve, the Kaplan-Meier analysis, and the Cox multivariable model were used for data analysis.

**Results:**

We identified 1,615 patients with T1-2N1M0 breast cancer, and 44.9% (*n* = 744) of them were treated with PMRT. With a median follow-up of 76 months, 46 (2.8%) recurrences, 96 (5.9%) deaths, and 80 (5.0%) breast cancer-related deaths occurred. The 5-year LRFS, DMFS, DFS, BCSS, and OS were 98.6%, 95.3%, 93.7%, 96.5%, and 96.0%, respectively. PMRT could not improve 5-year LRFS, DMFS, DFS, BCSS, and OS compared with non-PMRT neither before nor after PSM in the era of contemporary systemic treatment. ROC curve showed that the 8th pathological prognostic staging had better discriminative ability compared with the 7th anatomical staging [the area under the curve (AUC) 0.653 vs. 0.546, *P* < 0.001]. In the anatomical staging system, PMRT had comparable 5-year BCSS in comparison with non-PMRT both in stages IIA (97.4% vs. 96.8%, *P* = 0.799) and IIB (95.3% vs. 97.0%, *P* = 0.071). When stratified according to the pathological staging, PMRT was associated with better 5-year BCSS in stage IIB (97.1% vs. 90.7%, *P* = 0.039), while not in stages IA, IB, IIA, and IIIA. Multivariate analysis demonstrated that PMRT was a significantly protective factor for BCSS in stage IIB (HR 0.331, 95% CI: 0.100-0.967, *P* = 0.044).

**Conclusion:**

The new staging could better select high-risk patients with T1-2N1 breast cancer for radiotherapy compared with the 7th staging, and PMRT might be exempted except the 8th staging of IIB in the era of contemporary systemic therapy in this disease.

## 1. Introduction

Breast cancer ranks first in the prevalence of cancer among female patients and is the secondary cause of cancer-related mortality in the basis of 2021 prediction [1]. Patients with tumor size less than 5 cm and one to three involved axillary lymph nodes (LNs) (T1-2N1) are of the most interest to researchers among all cancer stages [2–6]. Surgery and chemotherapy are the cornerstone of this subtype; however, the effect of radiotherapy (RT) has not been well elaborated. Current clinical guidelines, such as the National Comprehensive Cancer Network guideline, strongly recommend postmastectomy radiotherapy (PMRT) for this patient subset [3]. The basis of their recommendation is derived from the result of the 2014 meta-analysis published in the Lancet that PMRT had significantly decreased locoregional recurrence (LRR) rate and cancer-related death in patients with 1-3 involved LNs [4]. However, most of the trials enrolled in this meta-analysis were completed before 1980s, when the RT technique and chemotherapy regimens were far from what it is now, and the LRR rate for the patients without PMRT was up to 30% at that time [5, 6]. Therefore, the value of PMRT in T1-2N1 breast cancer should be revalidated in the era of contemporary systematic treatment.

The traditional anatomical staging system has been extensively utilized for predicting prognosis and making treatment decision in breast cancer [7, 8]. However, the survival significance caused by molecular biomarker, such as estrogen receptor (ER), progesterone receptor (PR), and human epidermal growth factor-2 (HER2) make the anatomical staging unable to meet the trend of individualized treatment nowadays [9, 10]. Therefore, the 8th edition of American Joint Committee on Cancer (AJCC) pathological staging integrates ER, PR, HER2, and pathological grade into the anatomical staging, which exhibited better value of prognostic prediction in breast cancer [11, 12]. In the 7th AJCC staging, T1-2N1 breast cancer is divided into two substages (IIA and IIB), while the 8th AJCC staging classifies this group into five subtypes (IA-IIIA) [8, 13]. However, there were fewer studies exploring the effect of PMRT for T1-2N1 breast cancer patients in the basis of new pathological staging.

Therefore, we aimed to evaluate the effect of PMRT in the era of contemporary systematic treatment in T1-2N1 breast cancer. In addition, we further explored the value of prognostic prediction and RT administration decision of the 8th AJCC staging system in T1-2N1 breast cancer.

## 2. Materials and Methods

### 2.1. Patients

**Figure 1:**
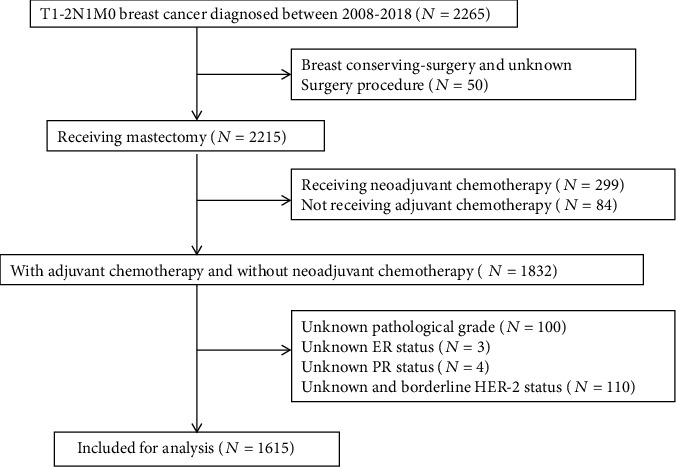
The selection flowchart of study population.

The patients were enrolled from Breast Cancer Information Management System (BCIMS) of West China Hospital, Sichuan University, which prospectively collects information on medical history, patient demographic, tumor characteristics, accessory examination, treatment, and follow-up, dating back to 1989. Patients meeting the following criterions were identified: (1) diagnosed between Jan 2008 and Dec 2018; (2) receiving radical or modified radical mastectomy; (3) pathologically diagnosed with pT1-2N1M0 invasive breast cancer; (4) receiving adjuvant chemotherapy; and (5) having detailed information on age, menopausal status, histological subtype, tumor stage, number of involved axillary LNs, ER status, PR status, HER2 status, pathological grade, the 7th AJCC anatomical stage, the 8th AJCC pathological stage, RT administration, endocrine therapy, anti-HER2 therapy, and follow-up. Male patients and patients aged ≤18 years, with bilateral breast cancer, and treated with neoadjuvant chemotherapy were excluded. The selection flowchart of the study population is presented in Figure 1.

The study was approved by the Biomedical Ethics Committee (Approval number: 2020427) of West China Hospital, Sichuan University. All patients in our database signed informed consent at initial diagnosis.

### 2.2. Treatments, Follow-Up, and Endpoints

All patients received radical mastectomy or modified radical mastectomy and adjuvant chemotherapy. Adjuvant chemotherapy protocols included anthracycline-based or taxane-based regimens and other regimens, such as cyclophosphamide, methotrexate, and fluorouracil (CMF) regimen, and oral chemotherapeutic drugs (capecitabine, S-1). RT administration was negotiated by the radiotherapy physicians and patients using three-dimensional conformal radiotherapy or intensity modulated radiotherapy techniques, with a prescribed dose of 46-50 grays in 25 fractions to the ipsilateral chest wall and infra- and supraclavicular regions. When tumors located in the inner side or central area of the breast, internal mammary LN region was included. Patients with hormone positive and HER2 overexpression received adjuvant endocrine therapy and anti-HER2 therapy, respectively, if economic conditions permitted. Follow-up information was collected by telephone calls, office visits, and emails. Follow-up protocols were as follows: once every 4 months in the first 3 years, once every six months during 4-5 years, and every year after 5 years.

The primary endpoint was breast cancer-specific survival (BCSS), calculated as the periods from surgery to the death of breast cancer. The secondary endpoints were locoregional-free survival (LRFS), calculated as the months from the start of surgery to the recurrence of ipsilateral chest wall, axillary, infra- or supraclavicular regions, or internal mammary LNs; distant metastasis-free survival (DMFS), from surgery to the metastasis of distant organs; disease-free survival (DFS), from surgical treatment to recurrence or metastasis or second primary breast cancer or any cause of death; and overall survival (OS), from surgery to any cause of death.

### 2.3. Statistical Analysis

We used the Chi-squared test to compare the difference of patient clinicopathological characteristics between the PMRT and non-PMRT groups. Propensity score matching (PSM) (1 : 1 nearest neighbor matching) was used to balance the patient characteristics including age at diagnosis, menopausal status, histological subtype, T stage, number of involved axillary LNs, ER, PR, HER2, grade, the 7th stage, and the 8th stage between the two groups. Kaplan-Meier analysis was utilized to calculate the differences and draw the survival curves of LRFS, BCSS, DMFS, DFS, and OS in patients receiving and not receiving PMRT. Cox multivariate hazards regression model was applied to identify the predictors of LRFS, BCSS, DMFS, DFS, and OS. The receiver operating characteristics (ROC) curve was utilized to discriminate the ability of predicting survival between the 7th and 8th AJCC staging. IBM SPSS 22.0 was used for analyzing and mapping. A *P* value <0.05 (two-tail) was considered to be statistically significant.

## 3. Results

### 3.1. Patient Characteristics and Treatment Information

A total of 1,615 patients with pT1-2N1M0 breast cancer were included in our institution.  Table 1 presents the detailed information of the study population before and after PSM. Before PSM, the proportion of patients aged <50 and ≥50 years were 58% (*n* = 936) and 42% (*n* = 679), respectively. Most of the patients were premenstrual (60.8%, *n* = 974), infiltrating ductal carcinoma (96.8%, *n* = 1,563), moderately/poorly differentiated (97.3%, *n* = 1,571), T2 stage (64.9%, *n* = 1,048), ER positive (76.1%, *n* = 1,229), PR positive (70.7%, *n* = 1,141), and one involved axillary LNs (62.4%, *n* = 1,008). A total of 44.9% (*n* = 744) of the patients received PMRT, while 55.1% (*n* = 871) of them were not treated with PMRT. Patients with younger age (*P* < 0.001), premenstrual (*P* < 0.001), T2 stage (*P* = 0.035), ER negative (*P* = 0.033), HER2 positive (*P* = 0.013), and three involved LNs (*P* < 0.001) were more likely to receive PMRT (Table 1).

When conducting PSM, after adjusting age, menopausal status, histological subtype, tumor stage, number of involved axillary LNs, ER status, PR status, HER2 status, pathological grade, the 7th staging system, and the 8th staging system, 624 pairs were completely matched between the PMRT and non-PMRT groups. Most of the patients were less than 50 years (61.3%, *n* = 765), premenstrual (63.6%, *n* = 794), infiltrating ductal carcinoma (96.6%, *n* = 1,205), moderately/poorly differentiated (97.4%, *n* = 1,215), T2 stage (64.4%, *n* = 804), ER positive (74.3%, *n* = 927), PR positive (69.6%, *n* = 868), and one involved axillary LNs (62.3%, *n* = 777). There was no significance in patient characteristics between the two groups after PSM (Table 1).

With regard to treatments, all patients received adjuvant chemotherapy. A total of 10.9% (*n* = 176), 14.4% (*n* = 233), 74% (*n* = 1,195), and 0.7% (*n* = 11) of the patients received anthracycline-based, taxane-based, anthracycline and taxane combination-based, and other regimens, respectively. In addition, 75% (*n* = 1,212) of the patients received endocrine therapy, accounting for 94% of ER positive patients (*n* = 1,229). Moreover, 12.6% (*n* = 204) of the patients received targeted therapy, accounting for 41.7% of HER2 overexpression patients (*n* = 470) (Table 1).

### 3.2. Survival Outcome and Prognostic Analysis for the Whole Cohort before and after PSM

**Figure 2:**
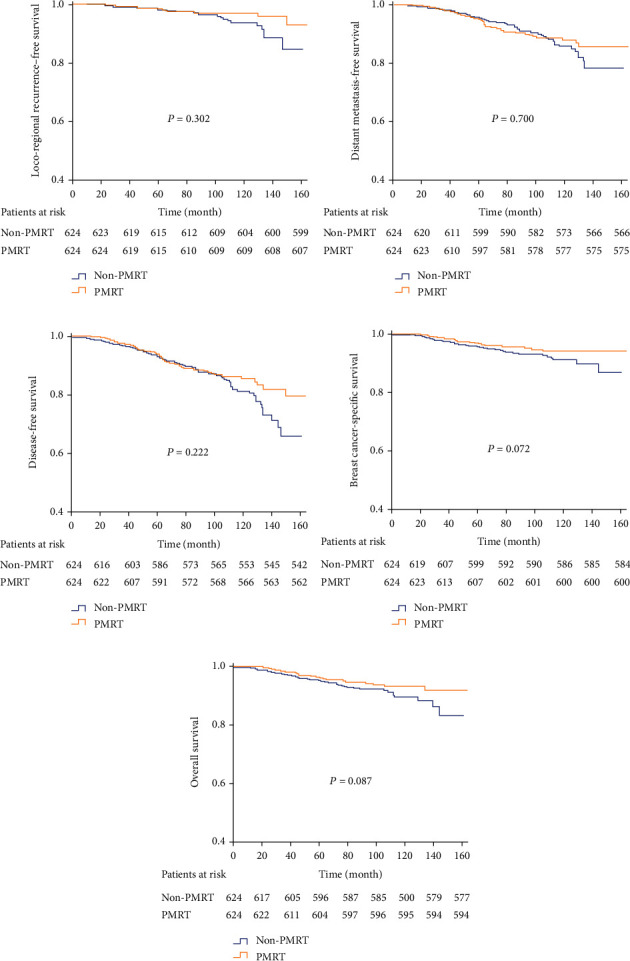
Kaplan-Meier curves of locoregional-free survival (LRFS) (a), distant metastasis-free survival (b), disease-free survival (DFS) (c), breast cancer-specific survival (BCSS) (d), and overall survival (OS) (e) in patients with or without postmastectomy radiotherapy (PMRT) after propensity score matching in the whole cohort.

With a median follow-up period of 76 months (4-164 months), 46 (2.8%) recurrences, 135 (8.4%) distant metastasis, 96 (5.9%) deaths, and 80 (5.0%) breast cancer-related deaths occurred. The 5-year LRFS, DMFS, DFS, BCSS, and OS were 98.6%, 95.3%, 93.7%, 96.5%, and 96.0% in the entire group. There was no significance of 5-year LRFS (98.6% vs. 98.5%, *P* = 0.267), DMFS (95.0% vs. 95.6%, *P* = 0.855), DFS (94.0% vs. 93.5%, *P* = 0.220), BCSS (97.0% vs. 96.1%, *P* = 0.112), and OS (96.5% vs. 95.5%, *P* = 0.089) in patients with and without PMRT before PSM (S3 Fig.). After balancing the patient characteristics between the two groups using PSM, PMRT could still not improve 5-year LRFS (98.5% vs. 98.5%, *P* = 0.302), DMFS (95.6% vs. 96.6%, *P* = 0.700), DFS (94.7% vs. 93.9%, *P* = 0.222), BCSS (97.3% vs. 96.0%, *P* = 0.072), and OS (96.8% vs. 95.5%, *P* = 0.087) compared with the non-PMRT group (Figure 2). Cox multivariate analysis showed that PMRT was not an independent predictor for LRFS [hazard ratio (HR) 0.751, 95% CI: 0.406-1.388, *P* = 0.361], DMFS (HR 0.983, 95% CI: 0.662-1.330, *P* = 0.721), DFS (HR 0.850, 95% CI: 0.627-1.152, *P* = 0.295), BCSS (HR 0.735, 95% CI: 0.461-1.171, *P* = 0.195), and OS (HR 0.779, 95% CI: 0.509-1.191, *P* = 0.249) (S1 Table and S2 Table).

### 3.3. Effect of PMRT on Survival according to the 7th and 8th AJCC Staging

**Figure 3:**
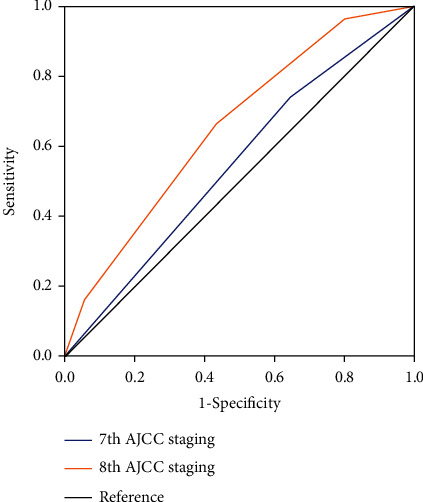
Time-dependent receiver operating characteristics analyses for assessing the discriminative value of breast cancer-specific survival between the 7th and 8th AJCC staging systems (predicted time: 164 months).

Among the patients in our study, 35.1% (*n* = 567) and 64.9% (*n* = 1048) had the 7th staging of IIA and IIB, while 19.4% (*n* = 314), 36.1% (*n* = 583), 22.4% (*n* = 362), 15.7% (*n* = 253), and 6.4% (*n* = 103) had the 8th staging of IA, IB, IIA, IIB, and IIIA. ROC analysis showed that the 8th pathological prognostic staging system [the area under the curve [(AUC) 0.653, 95% CI 0.595–0.710] had significant advantage in discriminating BCSS in comparison with the 7th staging system (AUC 0.546, 95% CI 0.484–0.609) (*P* < 0.001) with a predicted time of 164 months (Figure 3).

**Figure 4:**
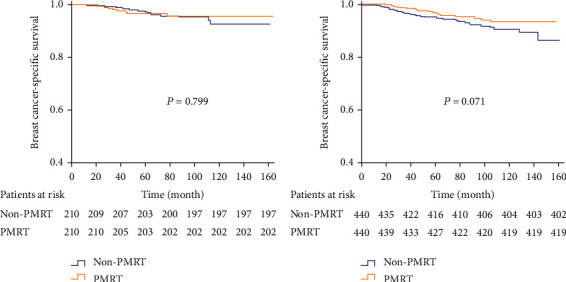
Kaplan-Meier curves for evaluating the value of PMRT according to the 7th anatomical staging system after PSM (A: stage IIA; B: stage IIB).

**Figure 5:**
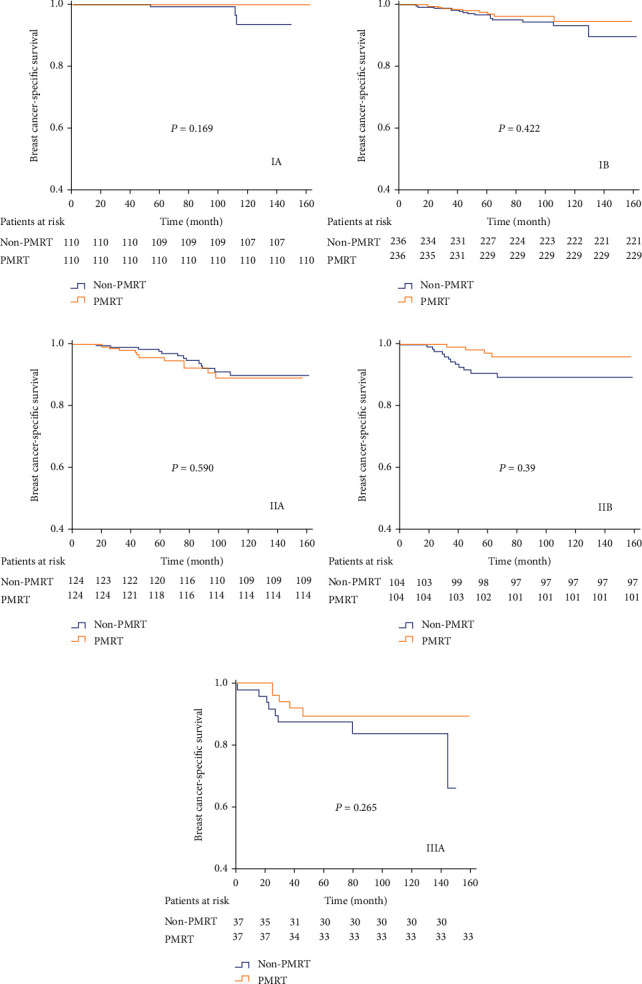
Kaplan-Meier curves for assessing the effect of PMRT on BCSS according to the 8th AJCC pathological prognostic staging system after PSM (A: stage IA; B: stage IB; C: stage IIA; D: stage IIB; E: stage IIIA).

In the anatomical staging, when performing PSM between the PMRT and non-PMRT cohorts, PMRT could not improve 5-year BCSS neither in stage IIA (97.4% vs. 96.8%, *P* = 0.799) nor IIB (95.3% vs. 97.0%, *P* = 0.071) compared with the non-PMRT group (Figure 4). Moreover, PMRT was not associated with better 5-year LRFS, DFS, and OS in the two substages in the 7th staging system. When stratified by the 8th AJCC staging, patients receiving PMRT had significantly better 5-year BCSS rate in stage IIB (97.1% vs. 90.7%, *P* = 0.039), while not in stages IA (100% vs. 99.2%, *P* = 0.169), IB (97.5% vs. 96.7%, *P* = 0.442), IIA (95.7% vs. 97.7%, *P* = 0.590), and IIIA (89.6% vs. 87.4%, *P* = 0.265) after PSM (Figure 5). Multivariate regression analysis showed PMRT was a significant predictor for BCSS only in stage IIB (HR 0.331, 95% CI: 0.100-0.967, *P* = 0.044) in the 8th AJCC staging (Table 2). In addition, PMRT was not associated with better 5-year LRFS (S4 Fig.), DFS (S5 Fig.), and OS (S6 Fig.) in all stages of the new pathological prognostic staging.

## 4. Discussion

In this study, we aimed to evaluate the value of PMRT in pT1-2N1M0 breast cancer patients with contemporary systematic treatment and further explored the guiding value of the anatomical staging and pathological prognostic staging systems on PMRT. Result demonstrated that PMRT could not improve 5-year LRFS, DMFS, DFS, BCSS, and OS compared with non-PMRT. The 8th AJCC staging could better predict the prognosis and guide the RT administration that patients with stage IIB could benefit from PMRT, while those with stages IA, IB, IIA, and IIIA could not.

The value of PMRT in T1-2N1 breast cancer has always been controversial. The large-scale meta-analysis in 2014 showed incremental benefit of PMRT on locoregional recurrence and breast cancer mortality, and the use of PMRT increased from 30.6% to 47.1% over years in T1-2N1 breast cancer patients [4, 14]. However, recent studies found that PMRT could not improve survival outcomes with modern systematic therapy [14–18]. Our result was consistent with theirs that patients receiving PMRT had no benefit on LRFS, DMFS, DFS, BCSS, and OS compared with those not receiving PMRT. The LRR rate of this study in the absence of PMRT was 3.3%, which was significantly lower than the studies included in 2014 meta-analysis (30%) [4, 19, 20]. This probably attributed to the progress of surgical techniques and modern chemotherapy regimens and anti-HER2 drugs [16]. In our study, 99.3% of the patients received taxane-based and/or anthracycline-based chemotherapy regimens, and only four patients received CMF regimen. Previous studies had demonstrated taxane-based and/or anthracycline-based regimens had better survival benefits compared with CMF regimen [16, 17]. In addition, all the patients in this study had negative surgical margins, and nearly half of the patients with HER2 overexpression received targeted therapy. Therefore, the administration of PMRT for T1-2N1 patients should be taken a cautious approach with such a lower LRR rate of 3.3% in the epoch of excellent systematic treatment. Let us look forward to the results of contemporary SUPREMO trial, an ongoing large-scale randomized controlled trial exploring the availability of PMRT in T1-2N1 breast cancer [21].

Previous studies have proven that the 8th AJCC staging had better prognostic value compared with anatomical staging in breast cancer [11, 12, 22]. However, few studies have evaluated the value of the new staging in predicting prognosis in T1-2N1 breast cancer, a disease with significant heterogeneity of clinical and histopathological behavior. In this study, the 8th AJCC pathological prognostic staging showed better discriminative value compared with the 7th staging in this disease (*P* < 0.001). However, patients with stage IIB had better survival than those with stage IIA in the 8th staging, which was different than the common knowledge and similar study from the Surveillance, Epidemiology, and End Results (SEER) database [23]. The possible reason was that racial disparities and the addition of molecular markers and pathological grade made the patients with stage IIB a better survival than those with stage IIA. Nonetheless, the new pathological staging indeed exhibited the heterogeneity of stage T1-2N1 disease and provided more precise prognostic information compared with anatomical staging.

Several recent studies, including our result, had demonstrated that PMRT showed no incremental survival benefit in T1-2N1 breast cancer patients receiving perfect contemporary systemic therapy [14–18]. Then, the selection of patients with high risk for PMRT in this highly heterogeneous disease appears to be essential. The 8th AJCC staging combining anatomical and pathological factors provides better choice of high-risk patients for PMRT [23, 24]. In our study, PMRT was not associated with improved survival in all stages of the 7th staging compared with non-PMRT in T1-2N1 breast cancer, while PMRT could improve BCSS in the new staging of IIB in this disease. Therefore, the new pathological prognostic staging had better value of selecting high-risk patients for receiving PMRT than the 7th staging. It is noteworthy that the 8th staging of IIB could benefit from PMRT, while stage IIIA could not in this study, which is inconsistent with the result that the higher stage is more likely to benefit from PMRT [25]. The possible explanation was that patients with stage IIIA were all triple negative breast cancer (ER, PR, and HER2) in our study population, which is highly invasive and more prone to have distant metastasis than other molecular types, and the value of locoregional RT cannot be reflected [26, 27]. Therefore, the administration of PMRT should be evaluated according to tumor and biologic characteristics. Our study recommended that PMRT could be administrated in stage IIB, while discreetly carried out in stage IIIA in view of new staging.

This study has several limitations that should be acknowledged. Firstly, this study is a retrospective study, and the intrinsic defects, such as selection bias and inconsistency, should not be neglected. Secondly, the baseline characteristics and treatment information between the PMRT and non-PMRT groups were not balanced, and patients receiving PMRT were more likely to have worse baseline characteristics, which could not represent the whole T1–2N1 breast cancer patients. Thirdly, the follow-up time of our study was short (medium: 76 months; range: 4-164 months), and long period is needed to validate the effect of PMRT in this disease. Finally, there were fewer patients with the 8th staging of IIIA, and more patients should be enrolled to evaluate the value of PMRT in this stage. Although this study has some limitations, we utilized new pathological staging to select the patients who need to receive PMRT in T1-2N1 breast cancer, which is in line with the trend of personalized treatment and reduce the economic burden for patients.

## 5. Conclusion

In conclusion, the 8th AJCC pathological prognostic staging had better value of guiding RT administration than the 7th anatomical staging in T1-2N1 breast cancer. PMRT might be exempted in the era of contemporary systemic therapy, except the 8th staging of IIB in this disease. Further studies should be conducted to evaluate the value of PMRT in T1-2N1 breast cancer.

## Figures and Tables

**Table 1:** Clinicopathological characteristics of the patients with and without PMRT before PSM and after PSM.

Variables		Before PSM (%)				After PSM (%)		
	Total	PMRT	Non-PMRT	*P*	Total	PMRT	Non-PMRT	*P*
Age (years)								
<50	936 (58.0)	470 (63.2)	466 (53.5)	<0.001	765 (61.3)	377 (60.4)	388 (63.2)	0.523
≥50	679 (42.0)	274 (36.8)	405 (46.5)		483 (38.7)	247 (39.6)	236 (37.8)	
Menopausal status								
Premenstrual	968 (60.0)	498 (66.9)	476 (57.6)	<0.001	794 (63.6)	396 (63.5)	398 (63.8)	0.993
Postmenstrual	637 (40.0)	246 (33.1)	395 (45.4)		454 (36.4)	228 (36.5)	226 (36.2)	
Histological subtype								
Infiltrating ductal carcinoma	1563 (96.8)	718 (96.5)	845 (97.0)	0.804	1205 (96.6)	604 (96.8)	601 (96.3)	0.969
Lobular carcinoma	13 (0.8)	7 (0.9)	6 (0.7)		11 (0.9)	5 (0.8)	6 (1.0)	
Other	39 (2.4)	19 (2.6)	20 (2.3)		32 (2.5)	15 (2.4)	17 (2.7)	
Pathological grade								
Well differentiated	44 (2.7)	16 (2.2)	28 (3.2)	0.100	33 (2.6)	16 (2.6)	17 (2.7)	0.821
Moderately differentiated	731 (45.3)	322 (43.3)	409 (47.0)		564 (45.2)	277 (44.4)	287 (46.0)	
Poorly differentiated/undifferentiated	840 (52.0)	406 (54.6)	434 (49.8)		651 (52.2)	331 (53.0)	320 (51.3)	
Tumor stage								
T1	567 (35.1)	241 (32.4)	326 (37.4)	0.035	444 (35.6)	207 (33.2)	237 (38.0)	0.076
T2	1048 (64.9)	503 (67.6)	545 (62.6)		804 (64.4)	417 (66.8)	387 (62.0)	
ER status								
Positive	1229 (76.1)	548 (73.7)	681 (78.2)	0.033	927 (74.3)	452 (72.4)	475 (76.1)	0.136
Negative	386 (23.9)	196 (26.3)	190 (21.8)		321 (25.7)	172 (27.6)	149 (23.9)	
PR status								
Positive	1141 (70.7)	522 (70.2)	619 (71.1)	0.69	868 (69.6)	419 (67.1)	449 (72.0)	0.065
Negative	474 (29.3)	222 (29.8)	252 (28.9)		380 (30.4)	205 (32.9)	175 (28.0)	
HER2 status								
Positive	470 (29.1)	239 (32.1)	231 (26.5)	0.013	386 (30.9)	201 (32.2)	185 (29.6)	0.741
Negative	1145 (70.9)	505 (67.9)	640 (73.5)		862 (69.1)	423 (67.8)	439 (70.4)	
Number of involved LN								
1	1023 (63.3)	382 (51.3)	641 (73.6)	<0.001	777 (62.3)	380 (60.9)	397 (63.6)	0.597
2	375 (23.2)	221 (29.7)	154 (17.7)		311 (24.9)	160 (25.6)	151 (24.2)	
3	217 (13.5)	141 (19.0)	76 (8.7)		160 (12.8)	84 (13.5)	76 (12.2)	
The 7th AJCC staging								
IIA	567 (35.1)	241 (32.4)	326 (37.4)	0.035	444 (35.6)	207 (33.2)	237 (38.0)	0.076
IIB	1048 (64.9)	503 (67.6)	545 (62.6)		804 (64.4)	417 (66.8)	387 (62.0)	
The 8th AJCC staging								
IA	314 (19.4)	135 (18.1)	179 (20.6)	0.434	250 (20.1)	111 (17.8)	139 (22.3)	0.267
IB	583 (36.1)	270 (36.3)	313 (35.9)		460 (36.9)	237 (38.0)	223 (35.7)	
IIA	362 (22.4)	162 (21.8)	200 (23.0)		248 (19.9)	121 (19.4)	127 (20.4)	
IIB	253 (15.7)	123 (16.5)	130 (14.9)		199 (15.9)	105 (16.8)	94 (15.1)	
IIIA	103 (6.4)	54 (7.3)	49 (5.6)		91 (7.2)	50 (8.0)	41 (6.6)	
Adjuvant chemotherapy regimen								
Anthracycline-based	176 (10.9)	124 (14.2)	52 (7.0)	<0.001	133 (10.7)	51 (8.2)	82 (13.1)	0.026
Taxane-based	233 (14.4)	148 (17.0)	85 (11.4)		162 (13.0)	77 (12.3)	85 (13.6)	
Anthracycline and taxane combination-based	1195 (74.0)	593 (68.1)	602 (80.9)		946 (75.7)	492 (78.8)	454 (72.8)	
Other	11 (0.7)	6 (0.7)	5 (0.7)		7 (0.6)	4 (0.6)	3 (0.5)	
Endocrine therapy								
Yes	1212 (75.0)	569 (76.5)	643 (73.8)	0.219	916 (73.4)	465 (74.5)	451 (72.3)	0.370
No	443 (25.0)	175 (23.5)	268 (26.2)		332 (26.6)	159 (25.5)	173 (27.7)	
Targeted therapy								
Yes	204 (12.6)	117 (15.7)	87 (10.0)	0.001	174 (13.9)	105 (16.8)	69 (11.1)	0.003
No	1411 (87.4)	627 (84.3)	784 (90.0)		1074 (86.1)	519 (83.2)	555 (88.9)	

**Table 2:** Cox multivariate analysis to determine the predictors of BCSS according to the 8th AJCC staging after propensity score matching.

Variables	BCSS		
	HR	95% CI	*P*
The 8th AJCC staging			
Stage IA			
Non-PMRT	1		
PMRT	0.022	0.001-330.123	0.441
Stage IB			
Non-PMRT	1		
PMRT	0.795	0.339-1.865	0.598
Stage IIA			
Non-PMRT	1		
PMRT	1.247	0.558-2.789	0.591
Stage IIB			
Non-PMRT	1		
PMRT	0.331	0.100-0.967	0.044
Stage IIIA			
Non-PMRT	1		
PMRT	0.553	0.178-1.713	0.304

BCSS, breast cancer-specific survival; PMRT, postmastectomy radiotherapy.

## Data Availability

The raw data supporting the conclusions of this article is available from the corresponding author on reasonable request.

## Supplementary Materials

Supplementary Materials The value of postmastectomy radiotherapy (PMRT) in T1-2N1M0 breast cancer remains unclear. Our study using real-world data demonstrated that the 8th edition of AJCC pathological prognostic staging system could better select high-risk patients with T1-2N1 breast cancer for radiotherapy compared with the 7th anatomical staging system, and PMRT might be exempted except the 8th staging of IIB in the era of contemporary systemic therapy in this disease. This is a file providing the supplementary tables and figures to support the study. S1 Table: Cox multivariate analysis for BCSS and OS of all the study population. S2 Table: Cox multivariate analysis for LRFS, DMFS, and DFS of all the patients. S3 Fig.: Kaplan-Meier curves of LRFS (A), DMFS (B), DFS (C), BCSS (D), and OS (E) in patients with or without PMRT before propensity score matching (PSM). S4 Fig.: Kaplan-Meier curves for assessing the effect of PMRT on LRFS stratified by the 8th AJCC staging after PSM (A: stage IA; B: stage IB; C: stage IIA; D: stage IIB; and E: stage IIIA). S5 Fig.: Kaplan-Meier curves for evaluating the value of PMRT on DFS according to the 8th AJCC pathological staging after PSM (A: stage IA; B: stage IB; C: stage IIA; D: stage IIB; and E: stage IIIA). S6 Fig.: Kaplan-Meier curves for assessment of effect of PMRT on OS according to the 8th AJCC staging after PSM (A: stage IA; B: stage IB; C: stage IIA; D: stage IIB; and E: stage IIIA).

## References

[1] Siegel R. L., Miller K. D., Fuchs H. E., Jemal A. (2021). Cancer statistics, 2021. *CA: a Cancer Journal for Clinicians*.

[2] Qi W. X., Cao L., Xu C., Zhao S., Chen J. (2020). Adjuvant regional nodal irradiation did not improve outcomes in T1-2N1 breast cancer after breast-conserving surgery: a propensity score matching analysis of BIG02/98 and BCIRG005 trials. *Breast*.

[3] The national comprehensive cancer network. NCCN clinical practice guidelines in oncology breast cancer. https://www.nccn.org/professionals/physician_gls/PDF/breast.pdf.

[4] McGale P., Correa C., Cutter D. (2014). Effect of radiotherapy after mastectomy and axillary surgery on 10-year recurrence and 20-year breast cancer mortality: meta-analysis of individual patient data for 8135 women in 22 randomised trials. *The Lancet*.

[5] Early Breast Cancer Trialists’ Collaborative Group (1995). Effects of radiotherapy and surgery in early breast cancer — An overview of the randomized trials. *The New England Journal of Medicine*.

[6] Overgaard M., Jensen M. B., Overgaard J. (1999). Postoperative radiotherapy in high-risk postmenopausal breast-cancer patients given adjuvant tamoxifen: Danish breast cancer cooperative group DBCG 82c randomised trial. *Lancet*.

[7] Edge S. B., Compton C. C. (2010). The American joint committee on cancer: the 7th edition of the AJCC cancer staging manual and the future of TNM. *Annals of Surgical Oncology*.

[8] Cserni G., Chmielik E., Cserni B., Tot T. (2018). The new TNM-based staging of breast cancer. *Virchows Archiv*.

[9] Bagaria S. P., Ray P. S., Sim M. S. (2014). Personalizing breast cancer staging by the inclusion of ER, PR, and HER2. *JAMA Surgery*.

[10] Park Y. H., Lee S. J., Cho E. Y. (2011). Clinical relevance of TNM staging system according to breast cancer subtypes. *Annals of Oncology*.

[11] Amin M. B., Greene F. L., Edge S. B. (2017). The eighth edition AJCC cancer staging manual: continuing to build a bridge from a population-based to a more "personalized" approach to cancer staging. *CA: a Cancer Journal for Clinicians*.

[12] Weiss A., Chavez-MacGregor M., Lichtensztajn D. Y. (2018). Validation study of the American joint committee on cancer eighth edition prognostic stage compared with the anatomic stage in breast cancer. *JAMA Oncology*.

[13] Hortobagyi G. N., Edge S. B., Giuliano A. (2018). New and important changes in the TNM staging system for breast cancer. *American Society of Clinical Oncology Educational Book*.

[14] Li F. Y., Lian C. L., Lei J. (2020). Real-world impact of postmastectomy radiotherapy in T1-2 breast cancer with one to three positive lymph nodes. *Annals of Translational Medicine*.

[15] Tam M. M., Wu S. P., Perez C., Gerber N. K. (2017). The effect of post-mastectomy radiation in women with one to three positive nodes enrolled on the control arm of BCIRG-005 at ten year follow-up. *Radiotherapy and Oncology*.

[16] McBride A., Allen P., Woodward W. (2014). Locoregional recurrence risk for patients with T1,2 breast cancer with 1-3 positive lymph nodes treated with mastectomy and systemic treatment. *International Journal of Radiation Oncology • Biology • Physics*.

[17] Muhsen S., Moo T. A., Patil S. (2018). Most breast cancer patients with T1-2 tumors and one to three positive lymph nodes do not need postmastectomy radiotherapy. *Annals of Surgical Oncology*.

[18] Ohri N., Haffty B. G. (2018). Is there a role for postmastectomy radiation (PMRT) in patients with T1-2 tumors and one to three positive lymph nodes treated in the modern era?. *Annals of Surgical Oncology*.

[19] Overgaard M., Hansen P. S., Overgaard J. (1997). Postoperative radiotherapy in high-risk premenopausal women with breast cancer who receive adjuvant chemotherapy. Danish breast cancer cooperative group 82b trial. *The New England Journal of Medicine*.

[20] Overgaard M., Nielsen H. M., Overgaard J. (2007). Is the benefit of postmastectomy irradiation limited to patients with four or more positive nodes, as recommended in international consensus reports? A subgroup analysis of the DBCG 82 b&c randomized trials. *Radiotherapy and Oncology*.

[21] Kunkler I. H., Canney P., van Tienhoven G., Russell N. S., MRC/EORTC (BIG 2-04) SUPREMO Trial Management Group (2008). Elucidating the role of chest wall irradiation in 'intermediate-risk' breast cancer: the MRC/EORTC SUPREMO trial. *Clinical Oncology (R Coll Radiol)*.

[22] Ibis K., Ozkurt S., Kucucuk S., Yavuz E., Saip P. (2018). Comparison of pathological prognostic stage and anatomic stage groups according to the updated version of the American joint committee on cancer (AJCC) breast cancer staging 8th edition. *Medical Science Monitor*.

[23] Wu S. G., Wang J., Lian C. L. (2020). Evaluation of the 8th edition of the American joint committee on cancer's pathological staging system in prognosis assessment and treatment decision making for stage T1-2N1 breast cancer after mastectomy. *Breast*.

[24] Wang S., Wen G., Tang Y. (2020). Effectiveness of the AJCC 8th edition staging system for selecting patients with T1-2N1 breast cancer for post-mastectomy radiotherapy: a joint analysis of 1986 patients from two institutions. *BMC Cancer*.

[25] Frasier L. L., Holden S., Holden T. (2016). Temporal trends in postmastectomy radiation therapy and breast reconstruction associated with changes in national comprehensive cancer network guidelines. *JAMA Oncology*.

[26] Lin N. U., Claus E., Sohl J., Razzak A. R., Arnaout A., Winer E. P. (2008). Sites of distant recurrence and clinical outcomes in patients with metastatic triple-negative breast cancer. *Cancer*.

[27] Yin L., Duan J. J., Bian X. W., Yu S. C. (2020). Triple-negative breast cancer molecular subtyping and treatment progress. *Breast Cancer Research*.

