# Supplementary material for: The Value of the 8th Edition of American Joint Committee on Cancer Pathological Prognostic Staging on the Selection of Postmastectomy Radiotherapy for T1–2N1 Breast Cancer
Source: J Oncol. 2022 Oct 25;2022:7550323. doi: 10.1155/2022/7550323 (PMC9626208; doi:10.1155/2022/7550323)

**Supplementary Materials**

**The value of the 8th edition of American Joint Committee on Cancer pathological prognostic staging on the selection of post-mastectomy radiotherapy for T1–2N1 breast cancer**

The value of post-mastectomy radiotherapy (PMRT) in T1-2N1M0 breast cancer remains unclear. Our study using real-world data demonstrated that the 8th edition of AJCC pathological prognostic staging system could better select high-risk patients with T1-2N1 breast cancer for radiotherapy compared with the 7th anatomical staging system, and PMRT might be exempted except the 8th staging of IIB in the era of contemporary systemic therapy in this disease. This is a file providing the supplementary tables and figures to support the study.

**Contents**

**S1 Table**. Cox multivariate analysis for BCSS and OS of all the study population.

**S2 Table**. Cox multivariate analysis for LRFS, DMFS, DFS of all the patients.

**S3 Fig**. Kaplan-Meier curves of LRFS (A), DMFS (B), DFS (C), BCSS (D), and OS (E) in patients with or without PMRT before propensity score matching (PSM).

**S4 Fig.** Kaplan-Meier curves for assessing the effect of PMRT on LRFS stratified by the 8th AJCC staging after PSM (A: stage IA; B: stage IB; C: stage IIA; D: stage IIB; E: stage IIIA).

**S5 Fig.** Kaplan-Meier curves for evaluating the value of PMRT on DFS according to the 8th AJCC pathological staging after PSM (A: stage IA; B: stage IB; C: stage IIA; D: stage IIB; E: stage IIIA).

**S6 Fig.** Kaplan-Meier curves for assessment of effect of PMRT on OS according to the 8th AJCC staging after PSM (A: stage IA; B: stage IB; C: stage IIA; D: stage IIB; E: stage IIIA).

**S1 Table**. Cox multivariate analysis for BCSS and OS of all the study population

| Variables | BCSS | | |  | OS | | |
| --- | --- | --- | --- | --- | --- | --- | --- |
|  | HR | 95%CI | P |  | HR | 95%CI | P |
| Age (years) |  |  |  |  |  |  |  |
| <50 | 1 |  |  |  | 1 |  |  |
| ≥50 | 0.877 | 0.429-1.790 | 0.718 |  | 0.790 | 0.415-1.503 | 0.472 |
| Menopausal status |  |  |  |  |  |  |  |
| Premenstrual | 1 |  |  |  | 1 |  |  |
| Postmenstrual | 1.649 | 0.807-3.370 | 0.170 |  | 2.113 | 1.107-4.036 | 0.023 |
| Number of involved LN |  |  |  |  |  |  |  |
| 1 | 1 |  |  |  | 1 |  |  |
| 2 | 1.292 | 0.771-2.167 | 0.331 |  | 1.44 | 0.908-2.282 | 0.121 |
| 3 | 1.163 | 0.595-2.275 | 0.659 |  | 1.091 | 0.576-2.066 | 0.788 |
| The 8th AJCC staging |  |  |  |  |  |  |  |
| IA | 1 |  |  |  | 1 |  |  |
| IB | 4.422 | 1.330-14.700 | 0.015 |  | 3.289 | 1.274-8.489 | 0.014 |
| IIA | 5.927 | 1.760-19.960 | 0.004 |  | 3.959 | 1.504-10.420 | 0.005 |
| IIB | 5.776 | 1.603-20.806 | 0.007 |  | 3.372 | 1.186-9.589 | 0.023 |
| IIIA | 10.66 | 2.677-42.461 | <0.001 |  | 5.43 | 1.725-17.086 | 0.004 |
| PMRT |  |  |  |  |  |  |  |
| No | 1 |  |  |  | 1 |  |  |
| Yes | 0.735 | 0.461-1.171 | 0.195 |  | 0.779 | 0.509-1.191 | 0.249 |
| Endocrine therapy |  |  |  |  |  |  |  |
| No | 1 |  |  |  | 1 |  |  |
| Yes | 0.796 | 0.440-1.441 | 0.452 |  | 0.642 | 0.377-1.0933 | 0.102 |
| Targeted therapy |  |  |  |  |  |  |  |
| No | 1 |  |  |  | 1 |  |  |
| Yes | 0.555 | 0.217-1.416 | 0.218 |  | 0.449 | 0.178-1.135 | 0.090 |
| BCSS, breast cancer specific survival; OS, overall survival; LN, lymph nodes; PMRT, post-mastectomy radiotherapy | | | | | | | |

**S2 Table**. Cox multivariate analysis for LRFS, DMFS, DFS of all the patients

| Variables | LRFS | | |  | DMFS | | |  | DFS | | |
| --- | --- | --- | --- | --- | --- | --- | --- | --- | --- | --- | --- |
|  | HR | 95%CI | P |  | HR | 95%CI | P |  | HR | 95%CI | P |
| Age (years) |  |  |  |  |  |  |  |  |  |  |  |
| <50 | 1 |  |  |  | 1 |  |  |  | 1 |  |  |
| ≥50 | 0.445 | 0.175-1.133 | 0.089 |  | 1.075 | 0.619-1.868 | 0.798 |  | 0.881 | 0.545-1.425 | 0.605 |
| Menopausal status |  |  |  |  |  |  |  |  |  |  |  |
| Premenstrual | 1 |  |  |  | 1 |  |  |  | 1 |  |  |
| Postmenstrual | 2.003 | 0.805-4.993 | 0.135 |  | 1.142 | 0.656-1.989 | 0.639 |  | 1.518 | 0.939-2.455 | 0.089 |
| Number of involved LN |  |  |  |  |  |  |  |  |  |  |  |
| 1 | 1 |  |  |  | 1 |  |  |  | 1 |  |  |
| 2 | 0.838 | 0.392-1.793 | 0.649 |  | 1.081 | 0.719-1.626 | 0.709 |  | 1.126 | 0.791-1.602 | 0.51 |
| 3 | 1.408 | 0.627-3.159 | 0.407 |  | 1.036 | 0.621-1.726 | 0.893 |  | 1.182 | 0.766-1.822 | 0.45 |
| The 8th AJCC staging |  |  |  |  |  |  |  |  |  |  |  |
| IA | 1 |  |  |  | 1 |  |  |  | 1 |  |  |
| IB | 5.054 | 0.646-39.549 | 0.123 |  | 4.623 | 1.830-11.678 | 0.001 |  | 3.566 | 1.768-7.191 | <0.001 |
| IIA | 11.313 | 1.490-85.916 | 0.019 |  | 5.966 | 2.335-15.243 | <0.001 |  | 4.040 | 1.972-8.280 | <0.001 |
| IIB | 8.184 | 0.992-67.520 | 0.051 |  | 6.643 | 2.492-17.704 | <0.001 |  | 3.962 | 1.843-8.519 | <0.001 |
| IIIA | 9.258 | 0.986-86.951 | 0.051 |  | 10.353 | 3.416-31.378 | <0.001 |  | 5.499 | 2.290-13.21 | <0.001 |
| PMRT |  |  |  |  |  |  |  |  |  |  |  |
| No | 1 |  |  |  | 1 |  |  |  | 1 |  |  |
| Yes | 0.751 | 0.406-1.388 | 0.361 |  | 0.983 | 0.662-1.330 | 0.721 |  | 0.850 | 0.627-1.152 | 0.295 |
| Endocrine therapy |  |  |  |  |  |  |  |  |  |  |  |
| No | 1 |  |  |  | 1 |  |  |  | 1 |  |  |
| Yes | 0.595 | 0.288-1.233 | 0.163 |  | 1.151 | 0.715-1.854 | 0.562 |  | 0.849 | 0.569-1.268 | 0.424 |
| Targeted therapy |  |  |  |  |  |  |  |  |  |  |  |
| No | 1 |  |  |  | 1 |  |  |  | 1 |  |  |
| Yes | 0.570 | 0.170-1.912 | 0.362 |  | 1.423 | 0.838-2.418 | 0.192 |  | 1.065 | 0.645-1.757 | 0.806 |
| LRFS, locoregional-free survival; DMFS, distant metastasis-free survival; DFS, disease free survival; LN, lymph nodes; PMRT, post-mastectomy radiotherapy | | | | | | | | | | | |

**S3 Fig**. Kaplan-Meier curves of LRFS (A), DMFS (B), DFS (C), BCSS (D), and OS (E) in patients with or without PMRT before PSM.

**
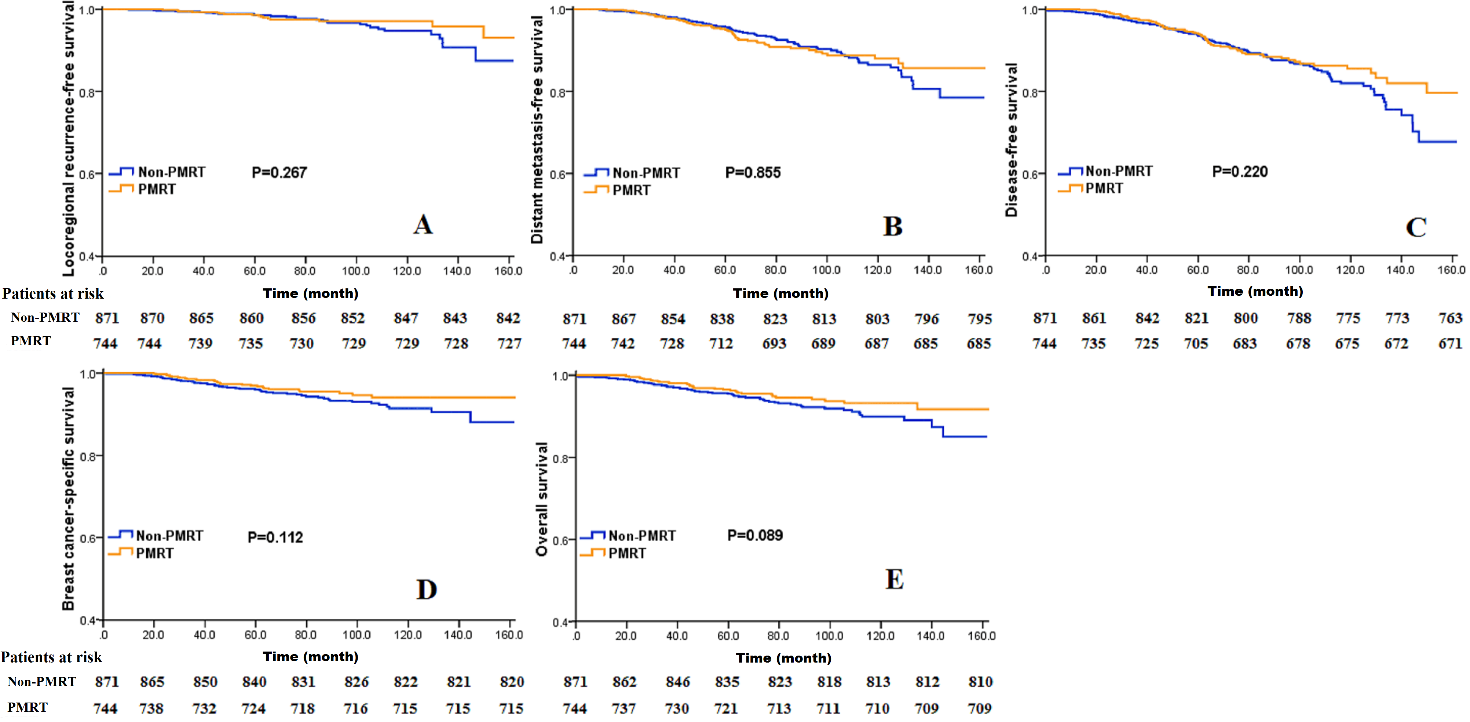
**

**S4 Fig.** Kaplan-Meier curves for assessing the effect of PMRT on LRFS stratified by the 8th AJCC staging after PSM (A: stage IA; B: stage IB; C: stage IIA; D: stage IIB; E: stage IIIA)


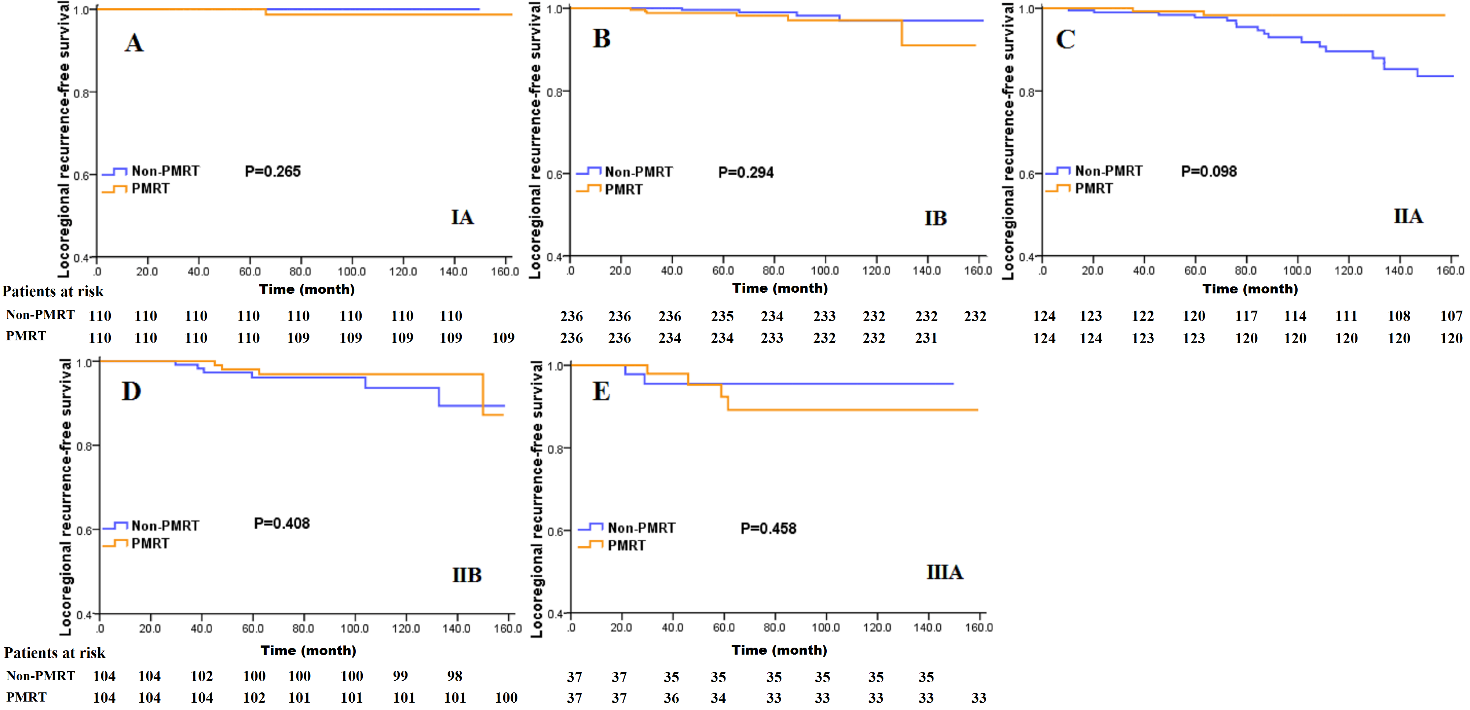


**S5 Fig.** Kaplan-Meier curves for evaluating the effect of PMRT on DFS according to the 8th AJCC pathological staging after PSM (A: stage IA; B: stage IB; C: stage IIA; D: stage IIB; E: stage IIIA).


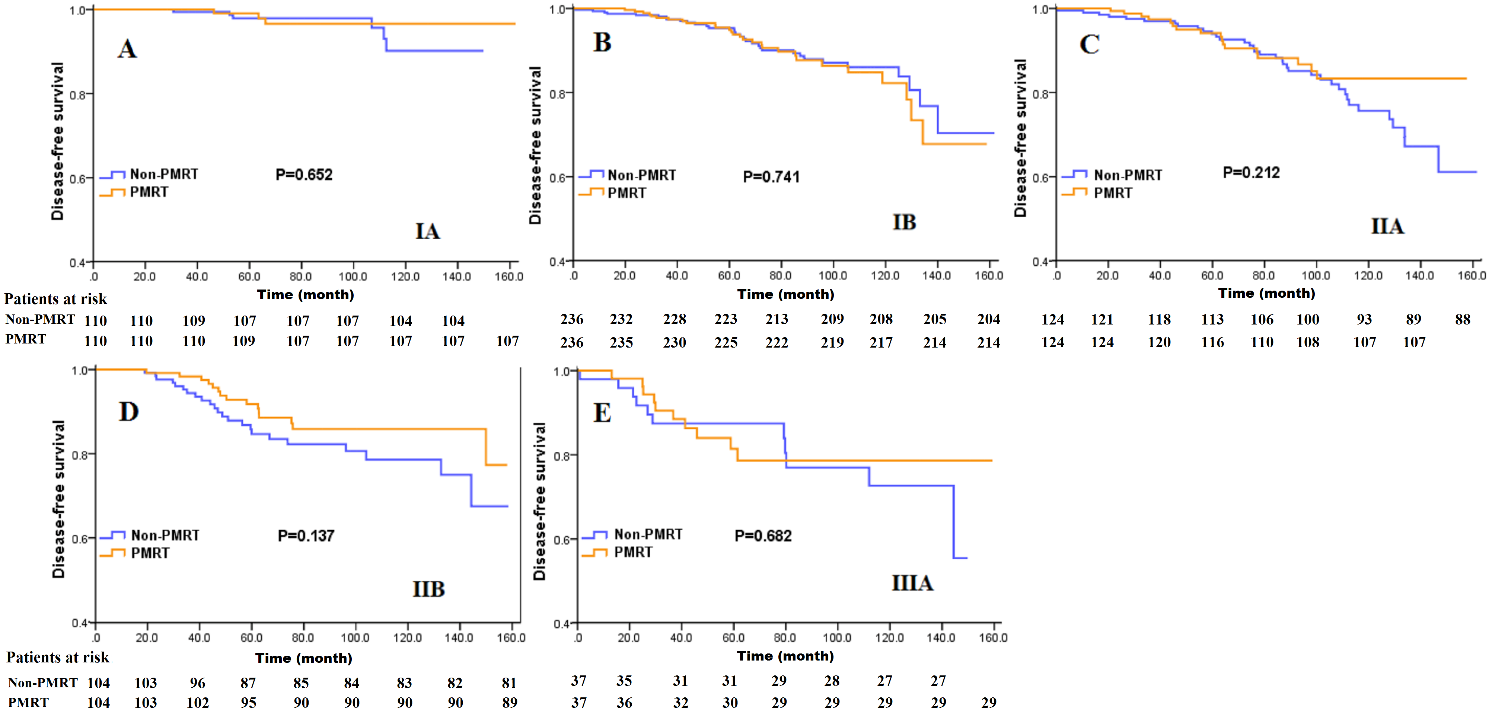


**S6 Fig.** Kaplan-Meier curves for assessment of effect of PMRT on OS according to the 8th AJCC staging after PSM (A: stage IA; B: stage IB; C: stage IIA; D: stage IIB; E: stage IIIA).


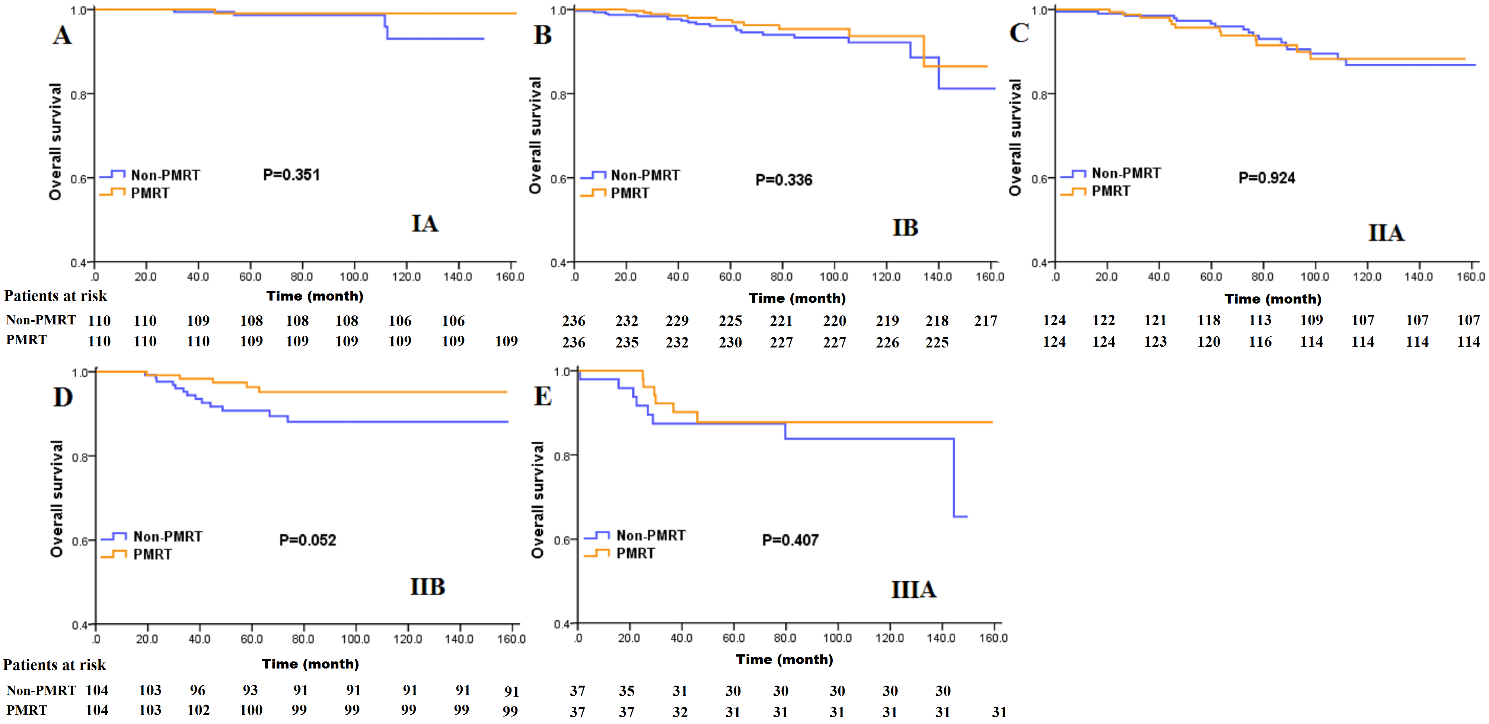

Supplement: Supplementary Materials — The value of postmastectomy radiotherapy (PMRT) in T1-2N1M0 breast cancer remains unclear. Our study using real-world data demonstrated that the 8th edition of AJCC pathological prognostic staging system could better select high-risk patients with T1-2N1 breast cancer for radiotherapy compared with the 7th anatomical staging system, and PMRT might be exempted except the 8th staging of IIB in the era of contemporary systemic therapy in this disease. This is a file providing the supplementary tables and figures to support the study. S1 Table: Cox multivariate analysis for BCSS and OS of all the study population. S2 Table: Cox multivariate analysis for LRFS, DMFS, and DFS of all the patients. S3 Fig.: Kaplan-Meier curves of LRFS (A), DMFS (B), DFS (C), BCSS (D), and OS (E) in patients with or without PMRT before propensity score matching (PSM). S4 Fig.: Kaplan-Meier curves for assessing the effect of PMRT on LRFS stratified by the 8th AJCC staging after PSM (A: stage IA; B: stage IB; C: stage IIA; D: stage IIB; and E: stage IIIA). S5 Fig.: Kaplan-Meier curves for evaluating the value of PMRT on DFS according to the 8th AJCC pathological staging after PSM (A: stage IA; B: stage IB; C: stage IIA; D: stage IIB; and E: stage IIIA). S6 Fig.: Kaplan-Meier curves for assessment of effect of PMRT on OS according to the 8th AJCC staging after PSM (A: stage IA; B: stage IB; C: stage IIA; D: stage IIB; and E: stage IIIA). [file 7550323.f1.docx]
